# Putative alternative polyadenylation (APA) events in the early interaction of *Salmonella enterica Typhimurium* and human host cells

**DOI:** 10.1016/j.gdata.2015.10.001

**Published:** 2015-10-24

**Authors:** Fabian Afonso-Grunz

**Affiliations:** Institute for Molecular BioSciences, Goethe University Frankfurt am Main, Frankfurt am Main, Germany; GenXPro GmbH, Frankfurt Biotechnology Innovation Center (FIZ), Frankfurt am Main, Germany

**Keywords:** Dual 3'Seq, Host-pathogen interaction, *Salmonella enterica*, Gene expression profiling, Alternative polyadenylation, Post-transcriptional regulation, Next‐generation sequencing

## Abstract

The immune response of epithelial cells upon infection is mediated by changing activity levels of a variety of proteins along with changes in mRNA, and also ncRNA abundance. Alternative polyadenylation (APA) represents a mechanism that diversifies gene expression similar to alternative splicing. T-cell activation, neuronal activity, development and several human diseases including viral infections involve APA, but at present it remains unclear if this mechanism is also implicated in the response to bacterial infections. Our recently published study of interacting *Salmonella enterica Typhimurium* and human host cells includes genome-wide expression profiles of human epithelial cells prior and subsequent to infection with the invasive pathogen. The generated dataset (GEO accession number: GSE61730) covers several points of time post infection, and one of these interaction stages was additionally profiled with MACE-based dual 3'Seq, which allows for identification of polyadenylation (PA) sites. The present study features the polyadenylation landscape in early interacting cells based on this data, and provides a comparison of the identified PA sites with those of a corresponding 3P-Seq dataset of non-interacting cells. Differential PA site usage of *FTL*, *PRDX1* and *VAPA* results in transcription of mRNA isoforms with distinct sets of miRNA and protein binding sites that influence processing, localization, stability, and translation of the respective mRNA. APA of these candidate genes consequently harbors the potential to modulate the host cell response to bacterial infection.

**Table:** 

Specifications	
Organism/cell line/tissue	*Homo sapiens*, HeLa-S3 *Salmonella enterica* subspecies I serotype *Typhimurium* strain SL1344
Sex	Female human cell line
Sequencer or array type	Illumina HiSeq2000 (1 × 50 base pair reads)
Data format	Raw and analyzed
Experimental factors	Infection assays were carried out with an MOI of 5. Total RNA isolates from HeLa and *S. enterica Typhimurium* cells were prepared after three different points of time (0.5, 4, and 24 h post infection, respectively) as well as from non-interacting cells.
Experimental features	The poly(A)^+^ and poly(A)^−^ fractions of interacting and non-interacting cells were used for distinct library preparation of interacting and non-interacting pathogen and host cells by deepSuperSAGE. One point of time post infection (0.5 h) was additionally prepared by MACE (Massive Analysis of cDNA Ends) as alternative tag-based library preparation method.
Consent	Not applicable
Sample source location	Not applicable

## 1 Direct link to deposited data.

http:/www.ncbi.nlm.nih.gov/geo/query/acc.cgi?acc=GSM1511911.

## 2 Introduction

Alternative polyadenylation (APA) is a common regulatory mechanism of gene expression that generates messenger RNAs (mRNAs) with distinct untranslated regions (UTRs) as well as coding sequences from one and the same gene. The resulting transcript isoforms may not only exhibit an altered coding potential, but also harbor a distinct set of cis-regulatory elements for microRNAs (miRNAs) and other non-coding RNAs (ncRNAs) as well as RNA-binding proteins (RBPs) that affects processing, localization, stability, and translation of the mRNA [1]. APA consequently adds to the complexity of eukaryotic transcriptomes similar to alternative splicing. Both polyadenylation and splicing are co-transcriptional processes, and the widespread APA of human introns suggests mutual interplays [2]. Polyadenylation (PA) sites within introns can result in conversion of an internal exon to a 3′ terminal exon or in usage of a 3′ terminal exon that is otherwise skipped. On top of this, debranched intron lariats can give rise to mirtrons, a widespread class of intron-derived miRNAs in animals that are produced by refolding of lariats during mRNA splicing [3], [4]. APA was shown to be implicated in T-cell activation, neuronal activity, development and several human diseases [5], [6] including viral infections [7]. Nonetheless, it remains unclear if APA is also involved in the response to bacterial infections.

Dual 3'Seq allows for reliable and comprehensive profiling of interacting pro- and eukaryotic cells with minimal sequencing efforts and without the need for prior fixation or physical disruption of the interaction [8]. Our recently published dataset of interacting *Salmonella enterica Typhimurium* and human epithelial cells comprises genome-wide expression profiles of the host cells prior and subsequent to infection with the invasive pathogen along with the corresponding prokaryotic transcriptomes. The data covers several points of time post infection (Table 1) and includes results from the two distinct 3′ end sequencing protocols MACE (Massive Analysis of cDNA Ends) and deepSuperSAGE (Serial Analysis of Gene Expression). Annotation to a combined reference comprising the operon-structured *S. enterica Typhimurium* as well as human genome sequence allowed for in silico separation of the interacting cells including quantification of polycistronic RNAs. However, our previous study was focused on the time-dependent expression profiles from pathogen and host cells based on the data generated by deepSuperSAGE. The potential of MACE to capture the polyadenylation landscape of eukaryotic cells was not exploited because of the missing information about PA site usage in non-interacting cells. Poly(A)-position profiling by sequencing (3P-Seq [9]) represents another technique for global detection of PA site usage besides MACE (reviewed in [10]), and a recent publication features a 3P-Seq dataset of cultured HeLa cells that were grown under similar conditions as in the present study [11]. We previously compared the high-confidence PA sites in the 3P-Seq dataset of mouse liver cells from Nam and colleagues with those obtained by MACE, and concluded that 3P-Seq and MACE provide similar results [12]. The deposited 3P-Seq dataset of non-interacting HeLa cells (GSM1268942) therefore served to complement our own data for identification of differentially used PA sites subsequent to infection.

*S. enterica Typhimurium* represents a human-virulent model organism that invades host cells through exploitation of their endocytosis machinery [13]. Once inside the cell, the pathogen starts intracellular replication, which requires formation of a unique cytoplasmic organelle, the *Salmonella*-containing vacuole (SCV) [14], [15]. The response of epithelial cells to bacterial invasion involves activation of signaling cascades that lead to expression of pro-inflammatory cytokines as well as increased production of reactive oxygen and nitrogen species (ROS and RNS, respectively) [16], [17]. ROS can promote pathogen elimination either by direct oxidative damage or via non-oxidative mechanisms such as stimulation of the inflammatory response and/or autophagy. At the same time, antioxidants such as ROS-scavengers protect the host cells from the damage caused by increased ROS production. The host cell immune response is mediated by changing activity levels of the involved proteins along with changes in mRNA, but also ncRNA abundance. The let-7 family of miRNAs was shown to post-transcriptionally regulate the mRNAs encoding IL-6 and IL-10 [18]. Exposure to bacterial lipopolysaccharides (LPS) resulted in downregulation of several members of the let-7 miRNA family in epithelial cells, which in turn relieved *IL-6* and *IL-10* from negative control. In addition to miRNA-mediated post-transcriptional regulation of target mRNAs, alternative splicing of certain mRNAs such as *HLA-B* in human cell lines [19], [20] and *IL-15* in transgenic mice [21] was also shown to affect the host response to bacterial invasion.

The present study addresses possible implications of APA as post-transcriptional regulation mechanism in the host cell response to infection by comparison of the identified polyadenylation landscape in early interacting HeLa-S3 cells with the deposited 3P-Seq dataset of non-interacting HeLa cells. Even though APA of the identified candidate genes remains to be validated, e.g. by qRT-PCR, at least some APA events seem to be tightly connected with the host cell response to bacterial infection.

## 3 Results

**Fig. 1:**
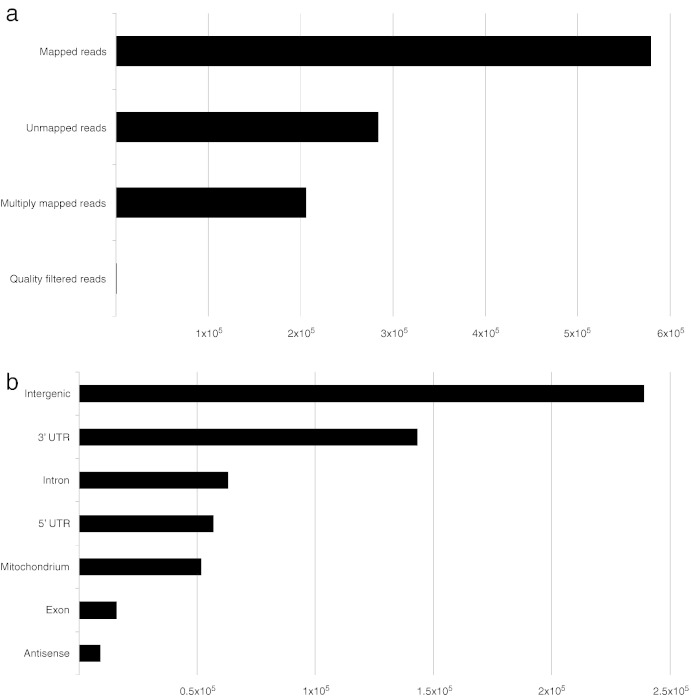
Mapping and annotation statistics of poly(A) tail positive reads from the poly(A)^+^ library of early interacting HeLa-S3 cells prepared with MACE. (a) The fraction of uniquely, ambiguously and unmapped mapped reads is shown along with the number of excluded reads during quality trimming. (b) The numbers of uniquely mapped reads that aligned to intergenic regions, the mitochondrion or in antisense direction of a protein-coding gene are shown together with the number of reads that mapped to the UTRs, introns and exons of mRNAs.

A little more than one million poly(A) tail positive reads remained after filtering and approximately half of these mapped to a unique locus within the human genome (Fig. 1a). As expected, a majority of the unambiguously mapped reads aligned to the 3′ UTR (Fig. 1b). Interestingly, even more poly(A) tail positive reads were located in intergenic regions, while approximately 10% mapped to introns or the 5′ UTR, respectively. The generated list of clusters (Supplementary Table S1) was screened for gene-specific PA sites present both in uninfected and early interacting cells. Further filtering resulted in 142 significantly differentially used PA sites (Bonferroni-adjusted p-value < 0.05) within 130 different genes. These genes were screened for gained or lost miRNA and protein binding sites as well as known interactions between the encoded proteins using APADB [12] and STRING [22].

**Fig. 2:**
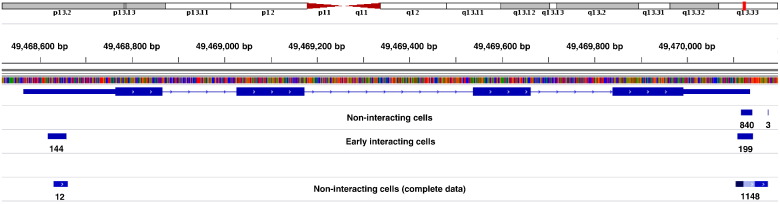
PA site usage of *FTL* in uninfected and early interacting host cells. The figure shows the region encoding FTL on human chromosome 19 together with the number and mapping position of poly(A) tail positive reads from uninfected (3P-Seq) and early interacting (MACE) HeLa cells. *FTL* comprises four exons, and three PA sites that are located within the UTRs. PA site usage in non-interacting cells is restricted to the 3′ UTR, while more than 40% of the mRNAs appear to be terminated within the 5′ UTR in early interacting cells. The complete 3P-Seq dataset from Nam and colleagues is additionally shown, and confirms the presence of the 5′ UTR isoform that is absent in the downsampled 3P-Seq dataset. The figure is based on an image from the Integrative Genomics Viewer [30].

Ferritin, light polypeptide (FTL) is involved in iron homeostasis, and the differential usage of PA sites from *FTL* in uninfected and early interacting host cells could contribute to the early response following bacterial infection. *FTL* exhibits three potential PA sites within HeLa cells (Fig. 2). Two of these give rise to transcript isoforms that solely differ in 3′ UTR length with ~ 40 nucleotides difference on average. The third putative PA site is located within the 5'UTR of *FTL*, thus pointing to a transcript isoform that comprises little more than 70 nucleotides. Since the slightly longer 3′ UTR isoform is only present in non-interacting cells in a vanishing low ratio (less than 1%), PA site usage primarily differs in between the shorter 3′ UTR and the very short 5′ UTR isoform. The shorter 3′ UTR isoform is preceded by a canonical poly(A) signal, and almost exclusively used in non-interacting cells. Early interacting cells, however, exhibit a prominent shift from this transcript variant to the 5′ UTR isoform. Compared to the canonical isoform, the 5′ UTR isoform appears to be almost equally abundant within these cells. Although being absent in the downsampled 3P-Seq data, the presence of this PA site is additionally confirmed by the complete 3P-Seq dataset (Fig. 2).

**Fig. 3:**
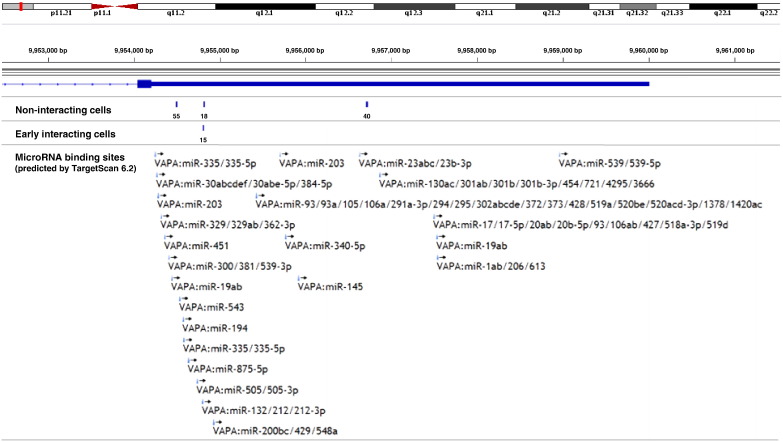
APA in the 3′ UTR of *VAPA* from uninfected and early interacting host cells. Depicted is the region that encodes the 3′ UTR of *VAPA* on human chromosome 18. The number of poly(A) tail positive reads is shown for each cluster along with the 3′ UTR miRNA binding sites of *VAPA*[31]. PA site usage differs in between three sites that give rise to mRNAs with several lost or gained miRNA binding sites. Please consult Fig. 2 for further details.

VAMP (vesicle-associated membrane protein)-associated protein A (VAPA) represents an endoplasmic reticulum (ER)-bound type IV membrane protein that regulates intracellular vesicle trafficking. The 3′ UTR of *VAPA* comprises numerous miRNA binding sites and exhibits three PA sites in non-interacting HeLa cells, while PA site usage in early interacting cells is restricted to a single site (Fig. 3). The 3′ UTR isoform that is also present in early interacting cells only constitutes 16% of all *VAPA* isoforms in uninfected cells. The most abundant isoform in non-interacting cells (almost 50% of all isoforms) is considerably shorter compared to the isoform that is also present in early interacting cells (~ 320 nucleotides difference on average), and lacks the binding sites for miR-543, miR-194, miR-335/335-5p, miR-875-5p, miR-505/505-3p as well as miR-132/212/212-3p. In contrast, the third and most distal PA site that is present in non-interacting cells gives rise to a 3′ UTR isoform that additionally harbors binding sites for miR-93/93a/105/106a/291a-3p/294/295/302abcde/372/373/428/519a/520be/520acd-3p/1378/1420ac, miR-23abc/23b-3p, miR-145, miR-200bc/429/548a, miR-203, and miR-340-5p.

**Fig. 4:**
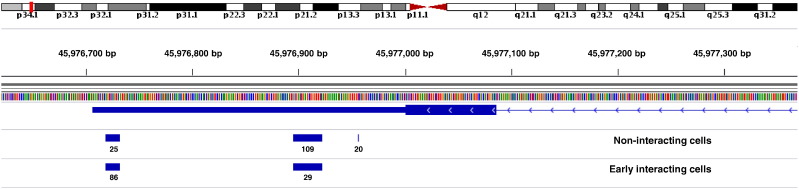
3′ UTR isoforms of *PRDX1* in uninfected and early interacting host cells. PRDX1 is encoded on the minus strand of human chromosome 1. The depicted region reveals three PA sites within the 3′ UTR of *PRDX1* from non-interacting cells. Two PA sites are also present subsequent to infection of the host cells, and the corresponding clusters exhibit an almost inversely proportional read distribution compared to non-interacting cells. Please consult Fig. 2 for further details.

Another gene that gives rise to mRNAs with distinct 3′ UTRs encodes Peroxiredoxin 1 (PRDX1). Only two out of three PA sites in non-interacting cells are also present subsequent to invasion of the cells (Fig. 4). With more than 70%, the most abundant 3′ UTR isoform in early interacting cells terminates at the most distal PA site. Conversely, more than 80% of all *PRDX1* isoforms are cleaved and polyadenylated at the two more proximal PA sites in uninfected cells. Since conserved miRNA binding sites are absent from the 3′ UTR of *PRDX1*, the region in between the two primarily used PA sites was screened for binding motifs of RBPs using RBPmap [23]. The predicted motifs mostly comprise binding sites of heterogeneous nuclear ribonucleoproteins (hnRNPs), followed by members of the serine/arginine (SR)-rich family of pre-mRNA splicing factors (SRSFs) along with other proteins that influence alternative splicing, transport and translation efficiency of *PRDX1* (Supplementary Table S2). The five highest ranking motifs are listed in Table 2 together with their associated RBPs.

## 4 Discussion

The underlying data for comparison of the identified polyadenylation landscape in interacting cells with the 3P-Seq dataset of non-interacting cells was processed with distinct parameters to level the differences between the two employed methods. Poly(A) site supporting reads were required to have at least ten or five 3′-terminal adenine bases for MACE and 3P-Seq, respectively. In general, detection of false positive PA sites due to inadvertent priming of homopolymeric adenosine stretches within the mRNAs is minimized by an increasing number of required 3′-terminal adenine bases. Given a read length of 50 nucleotides for MACE, a fifth of each read had to represent 3′-terminal adenine bases to be considered as poly(A) site supporting read. Since 3P-Seq circumvents conventional oligo(dT) priming during library preparation (hybridization of the oligo(dT) is followed by RNase H digestion), and given that the 3P-Seq reads from HeLa only comprise 36 nucleotides, the required five 3′-terminal adenine bases represent an accordingly stringent threshold that adds up to almost a sixth of the whole read.

The relatively high amount of available poly(A) site supporting 3P-Seq reads from non-interacting HeLa cells allowed for quality-trimming with an increased FASTQ Sanger quality score as threshold. The remaining set of high quality reads was subsequently downsampled for better comparability of the two datasets. MACE employs GenXPro's TrueQuant technology for PCR-bias free amplification, which allows for generation of completely unbiased transcriptome profiles. The data generated by 3P-Seq, on the other hand, is prone to PCR amplification bias. While this impeded a direct comparison of the read numbers from identified clusters within the two conditions, APA events were deduced by comparison of the ratios between a given cluster and all other clusters of that gene within each condition.

Iron is required by a wide variety of intracellular bacterial pathogens to achieve full virulence, and the availability of iron within the cytoplasm of host cells is regulated by a complex regulatory network that includes *FTL* and several other genes [24]. The 5′ UTR isoform of *FTL* harbors an iron-responsive element (IRE) that is bound by aconitase (ACO1), a bifunctional iron-sulfur protein that acts as an RBP when cells are iron-depleted. Binding of ACO1 to the IRE results in translational repression of the mRNA encoding FTL, thereby inhibiting assembly of new ferritin proteins, which in turn increases the labile iron pool over time [25]. Once the labile iron pool is restored to according levels, ACO1 dissociates from the IRE, and translation of *FTL* is derepressed. The shift from the canonical 3′ UTR isoform in non-interacting cells to the very short 5′ UTR isoform subsequent to bacterial invasion suggests two possible scenarios. Given that the 5′ UTR isoform is not preceded by any poly(A) signal, this isoform could represent a degradation intermediate that is not digested right away due to IRE-bound ACO1. This would imply that the 3′ end of the protein-shielded sequence is subject to transient polyadenylation within the cytoplasm to facilitate further degradation. While this represents a common mechanism in prokaryotes, cytosolic polyadenylation was only relatively recently shown to contribute to RNA degradation in humans [26]. The presence of degradation intermediates would indicate reduced levels of the labile iron pool caused by the increased iron consumption that arises from additional uptake of iron into intracellular bacteria. Degradation of *FTL* could thus help to compensate for this loss via inhibition of ferritin assembly. On the other hand, this isoform could also represent an alternatively polyadenylated transcript variant despite the lack of a preceding poly(A) signal. Since this isoform solely covers the ACO1 binding site with a few flanking nucleotides it could probably act as a scavenger for ACO1 that alters the regulatory network of iron homeostasis by derepression of IREs in other transcripts.

SCV biogenesis is initiated by specific interactions of internalized *Salmonella* with the early endocytic network of host cells [15], and maturation of the SCV requires acquisition as well as exclusion of specific late endocytic markers [14]. According to Brumell and colleagues, invasion of epithelial cells is followed by rapid and transient interactions of the SCV with early endosomes within the first 5 min. Further inter-organellar interactions of the SCV in the following 90 min mediate the delivery of certain endocytic markers, which finally uncouples the SCV from the endocytic pathway of the host. VAPA and VAPB are closely related and interact with FFAT-containing proteins such as STARD3 and STARD3NL during inter-organellar interactions of the ER with late endosomes [27]. VAPA is additionally involved in intraluminal vesicle (ILV) formation during endosome maturation, and it has been estimated that approximately half of all early and almost all late endosomes are in contact with the ER [28]. This raises the possibility that VAPA is at least indirectly affected by SCV biogenesis. The fact that PA site usage in early interacting cells is restricted to a single site that generates a 3′ UTR isoform with comprehensively altered miRNA binding capacities suggests an altered post-transcriptional regulation of *VAPA* in response to bacterial invasion. The 3′ UTR isoform that is present in early interacting cells lacks the binding sites for six miRNA families compared to the more distal isoform in non-interacting cells, while it harbors additional binding sites for six miRNA families compared to the more proximal isoform.

PRDX1 participates in redox regulation of the cell, which is especially important in the context of bacterial invasion. In infected HeLa cells, *PRDX1* exhibits a prominent shift to more distal PA sites, thus providing additional binding sites for several RBPs involved in regulation of alternative splicing such as hnRNPs and SRSFs (Table 2, Supplementary Table S2). The highest ranking RBP motif in the region between the two major PA sites of *PRDX1* recruits MATR3, an inner nuclear matrix protein that stabilizes mRNAs upon binding and that additionally binds to small ncRNAs involved in splicing. Against this backdrop, alternative PA site usage in non-interacting and infected cells could likely affect alternative splicing mechanisms. According to Ensembl [29], *PRDX1* encodes five alternative splicing isoforms and an additional ncRNA that lacks an open reading frame. Similar to the interplay between intronic polyadenylation and alternative splicing that affects the terminal exon of many human genes [2], APA within the 3′ UTR of *PRDX1* might result in altered splicing patterns during co-transcriptional processing of the transcript. Besides their role in alternative splicing, several of the identified RBPs are involved in transport and translation of mRNAs. The additional binding sites in distal 3′ UTR isoforms from infected HeLa cells could consequently also influence localization and translation efficiency of *PRDX1*.

## 5 Conclusions

The published dual 3'Seq dataset of interacting *S. enterica Typhimurium* and human host cells provides insights into the time-dependent and pathogenicity-related gene expression of the prokaryote along with corresponding changes in the transcriptome of host cells. The eukaryotic expression profiles include genome-wide abundance levels not only for protein-coding transcripts, but also for ncRNAs such as miRNA precursors. Information on PA site usage can additionally be inferred from MACE-based dual 3'Seq as shown here for the sequenced poly(A)^+^ library of early interacting HeLa cells.

The comparison of the identified polyadenylation landscape in early interacting cells with the published dataset of non-interacting cells determined by 3P-Seq indicates several significantly differentially used PA sites, and some of these suggest that APA might contribute to the complex regulatory network that governs the immune response of epithelial cells. APA of *PRDX1* results in transcription of mRNA isoforms with distinct sets of miRNA binding sites, while PA site usage in *VAPA* is likely to influence alternative splicing. The putative PA site in the 5′ UTR of *FTL* could give rise to a transcript isoform that alters the regulatory network of iron homeostasis by scavenging of ACO1. Taken together, these genes provide promising targets for further research of APA, especially in the context of bacterial infections, where the involvement of APA remains to be elucidated.

## 6 Materials and methods

### 6.1 Cell culturing, infection assays and library preparation

Cell culturing and infection assays were carried out with the HeLa-S3 cell line from LGC standards (ATCC CCL-2.2) and the *S. enterica* subspecies I serotype Typhimurium strain SL1344 as described in [8]. HeLa-S3 cells were infected at an MOI of 5, and total RNA was isolated from non-interacting and infected cells after three points of time p.i. (Table 1). The poly(A)^+^ and poly(A)^−^ fractions of the isolates were used for distinct library preparation via dual 3'Seq. Briefly, total RNA isolates were size-selected subsequent to DNase I digestion of DNA remnants in the isolates. Following rRNA depletion, the RNA was split into the poly(A)^+^ and poly(A)^−^ fraction by oligo(dT) capture to separate the polyadenylated and functional mRNAs of eukaryotic cells from the non-polyadenylated transcripts that represent the functional transcriptome of prokaryotes. After in-vitro polyadenylation of the poly(A)^−^ fraction, both fractions were reverse-transcribed using an anchored, biotinylated oligo(dT) primer. The generated cDNA was fragmented according to two established 3′ transcriptome profiling techniques. DeepSuperSAGE tags were generated via cleavage of RNAs by the anchoring enzyme NlaIII and subsequent digestion using EcoP15I, while MACE involved random fragmentation for generation of tags. 3′ fragments were enriched by binding to a streptavidin matrix and ligated to a sequencing adaptor. Adaptor-ligated fragments were PCR-amplified using GenXPro's TrueQuant technology for PCR-bias free amplification, PAGE-purified, and finally sequenced on the Illumina HiSeq2000 platform.

### 6.2 Identification of alternative polyadenylation events in early interacting HeLa cells

The sequenced poly(A)^+^ library of early interacting HeLa cells prepared with MACE was filtered for poly(A) site supporting reads with at least ten 3′-terminal adenine bases. The remaining reads were quality-trimmed (discarding nucleotides with a FASTQ Sanger quality score below 16). Trimmed reads comprising less than 20 nucleotides were excluded from the dataset to ensure reliable mapping results. Subsequent to clipping of the potential poly(A) tail, trimmed reads were mapped to hg19 using the short read mapper Novoalign (Novocraft Technologies, http://novocraft.com) with default parameter settings. Clustering of the mapped reads and annotation of the identified clusters was performed as described in [12].

The deposited 3P-Seq dataset (GSM1268942) was used to complement our own data for identification of differentially used PA sites within early interacting HeLa cells. The deposited reads were quality-trimmed (discarding nucleotides with a FASTQ Sanger quality score below 35 as well as trimmed reads < 20 nucleotides), and subsequently reverse complemented. The remaining reads were filtered for poly(A) site supporting reads with at least five 3′-terminal adenine bases, and subsequently downsampled to a comparable sequencing depth with respect to the poly(A)^+^ library of early interacting HeLa cells that was prepared with MACE. Clipping of the potential poly(A) tail, mapping, clustering and annotation of identified clusters was performed as described for the MACE library. In order to identify overlapping clusters for analysis of differentially used PA sites in uninfected and interacting HeLa cells, the mode of each cluster ± 10 nucleotides was compared between the two conditions (Supplementary Table S1). Subsequent statistical testing for condition-specific APA was carried out with Fisher's exact test followed by correction for multiple comparisons according to Bonferroni.

The following are the supplementary data related to this article.

Supplementary Table S1 Summary of the identified clusters for comparison of PA site usage in uninfected and early interacting HeLa cells. PA sites present in one of the two conditions are listed with the respective gene symbols and genomic coordinates. The number of reads in each cluster from a given gene is shown along with the number of reads in all other clusters from that gene for 3P-Seq and MACE, respectively. Statistical significance of the condition-specific ratios of these clusters according to Fisher's exact test is indicated by the respective p-values. PA sites present in both conditions or with a Bonferroni-adjusted p-value of less than 0.05 are additionally indicated by Booleans.

Supplementary Table S2 Summary of the predicted RBP binding motifs in the 3′ UTR of *PRDX1* according to RBPmap. The genomic coordinates of the screened region are listed along with the applied calculation parameters. Please consult Table 2 for further details.

## Figures and Tables

**Table 1:** Total RNA isolates were prepared from interacting cells at the listed points of time post infection (p.i.) as well as from non-interacting cells. All isolates were subject to deepSuperSAGE-coupled dual 3'Seq library preparation, while one point of time p.i. (0.5 h) was additionally prepared with MACE.

Interaction stage	Point of time (p.i.)	Available dual 3'Seq data
Non-interacting cells	Prior to infection	deepSuperSAGE
Early interaction	½ hour	MACE and deepSuperSAGE
Mid-level interaction	4 hours	deepSuperSAGE
Late interaction	24 hours	deepSuperSAGE

**Table 2:** List of the five highest ranking RBP target motifs in the 3′ UTR of *PRDX1*. The region between the two major PA sites of PRDX1 was screened for binding motifs with RBPmap using default parameters with high stringency levels. The identified k-mers are listed along with the respective genomic coordinates, Z-scores, and p-values.

Binding motif	Associated RPB	Genomic coordinate	K-mer	Z-score	p-Value
maucuur	MATR3	Chr1 - 45,976,737	aaacuug	3.681	1.16E-04
guaguagu	HNRNPA1	Chr1 - 45,976,778	guauuagu	3.623	1.46E-04
grhuuaa	ZCRB1	Chr1 - 45,976,742	guauuaa	3.325	4.42E-04
gguaguag	HNRNPA2B1	Chr1 - 45,976,776	auuaguag	3.203	6.80E-04
yywcwsg	SRSF5	Chr1 - 45,976,864	cuacagg	3.011	1.30E-03
