# Supplementary material for: Putative alternative polyadenylation (APA) events in the early interaction of Salmonella enterica Typhimurium and human host cells
Source: Genom Data. 2015 Oct 24;6:222–7. doi: 10.1016/j.gdata.2015.10.001 (PMC4664775; doi:10.1016/j.gdata.2015.10.001)
Supplement: Supplementary Table S2 — Summary of the predicted RBP binding motifs in the 3′ UTR of PRDX1 according to RBPmap. The genomic coordinates of the screened region are listed along with the applied calculation parameters. Please consult Table 2 for further details. [file mmc2.pdf]

Predictions for sequence: chr1:45976719-45976900:-
Calculation parameters:
Genome: Human (hg19)
Selected motifs: All Human/Mouse motifs
Stringency level: High
Conservation filter: On
==

Protein: CUG-BP(Hs/Mm)

Sequence Position	Genomic Coordinate	Motif	K-mer	Z-score	P-value
107	chr1:45976794	ugcug	uacug	1.707	4.39E-02
119	chr1:45976782	ugcug	ugaug	1.664	4.81E-02

Protein: DAZAP1(Hs/Mm)

Sequence Position	Genomic Coordinate	Motif	K-mer	Z-score	P-value
74	chr1:45976827	uagkwwr	uaggaau	2.545	5.46E-03

Protein: HNRNPA1(Hs/Mm)

Sequence Position	Genomic Coordinate	Motif	K-mer	Z-score	P-value
123	chr1:45976778	guaguagu	guauuagu	3.623	1.46E-04

Protein: HNRNPA2B1(Hs/Mm)

Sequence Position	Genomic Coordinate	Motif	K-mer	Z-score	P-value
125	chr1:45976776	gguaguag	auuaguag	3.203	6.80E-04

Protein: HNRNPC(Hs/Mm)

Sequence Position	Genomic Coordinate	Motif	K-mer	Z-score	P-value
145	chr1:45976756	huuuuuk	aucuuuu	2.697	3.50E-03

Protein: HNRNPCL1(Hs/Mm)

Sequence Position	Genomic Coordinate	Motif	K-mer	Z-score	P-value
145	chr1:45976756	huuuuuk	aucuuuu	2.673	3.76E-03

Protein: HNRNPF(Hs/Mm)

Sequence Position	Genomic Coordinate	Motif	K-mer	Z-score	P-value
44	chr1:45976857	gggug	gggug	1.676	4.69E-02

Protein: HNRNPU(Hs/Mm)

Sequence Position	Genomic Coordinate	Motif	K-mer	Z-score	P-value
10	chr1:45976891	uguauug	uguggug	2.397	8.26E-03
122	chr1:45976779	uguauug	uguauua	2.937	1.66E-03
158	chr1:45976743	uguauug	uguauua	2.651	4.01E-03

Protein: HuR(Hs/Mm)

Sequence Position	Genomic Coordinate	Motif	K-mer	Z-score	P-value
109	chr1:45976792	uukruuu	cugguuu	1.723	4.24E-02

Protein: MATR3(Hs/Mm)

Sequence Position	Genomic Coordinate	Motif	K-mer	Z-score	P-value
144	chr1:45976757	maucuur	aaucuuu	2.139	1.62E-02
164	chr1:45976737	maucuur	aaacuug	3.681	1.16E-04

Protein: MBNL1(Hs/Mm)

Sequence Position	Genomic Coordinate	Motif	K-mer	Z-score	P-value
12	chr1:45976889	ygcuky	uggugu	1.745	4.05E-02
62	chr1:45976839	ygcuky	uucuuc	2.098	1.80E-02
66	chr1:45976835	ygcuky	uccuuu	2.137	1.63E-02
93	chr1:45976808	ygcuky	cguugu	2.098	1.80E-02
110	chr1:45976791	ygcuky	ugguuu	2.343	9.56E-03
119	chr1:45976782	ygcuky	ugaugu	2.049	2.02E-02

Protein: PCBP3(Hs/Mm)

Sequence Position	Genomic Coordinate	Motif	K-mer	Z-score	P-value
32	chr1:45976869	uuuycc	cuuucc	1.841	3.28E-02

Protein: PTBP1(Hs/Mm)

Sequence Position	Genomic Coordinate	Motif	K-mer	Z-score	P-value
17	chr1:45976884	ucuu	ucuu	2.523	5.82E-03
31	chr1:45976870	ucuu	ccuu	1.955	2.53E-02
35	chr1:45976866	ucuu	uccu	2.099	1.79E-02
59	chr1:45976842	ucuu	ccuu	2.171	1.50E-02
60	chr1:45976841	cucucu	cuuucu	2.045	2.04E-02
63	chr1:45976838	ucuu	ucuu	2.658	3.93E-03
64	chr1:45976837	cucucu	cuuccu	1.673	4.72E-02
66	chr1:45976835	ucuu	uccu	2.099	1.79E-02
67	chr1:45976834	ucuu	ccuu	2.099	1.79E-02
124	chr1:45976777	ucuu	uauu	1.955	2.53E-02
146	chr1:45976755	ucuu	ucuu	2.73	3.17E-03
148	chr1:45976753	ucuu	uuuu	2.171	1.50E-02
160	chr1:45976741	ucuu	uauu	2.171	1.50E-02
166	chr1:45976735	ucuu	acuu	2.486	6.46E-03
179	chr1:45976722	ucuu	ccuu	2.207	1.37E-02

Protein: QKI(Hs/Mm)

Sequence Position	Genomic Coordinate	Motif	K-mer	Z-score	P-value
141	chr1:45976760	acuaay	auuaau	2.449	7.16E-03

Protein: RALY(Hs/Mm)

Sequence Position	Genomic Coordinate	Motif	K-mer	Z-score	P-value
146	chr1:45976755	uuuuuub	ucuuuug	2.78	2.72E-03

Protein: RBM28(Hs/Mm)

Sequence Position	Genomic Coordinate	Motif	K-mer	Z-score	P-value
127	chr1:45976774	gwguagd	uaguaga	1.761	3.91E-02
150	chr1:45976751	gwguagd	uuguagu	1.69	4.55E-02

Protein: RBM38(Hs/Mm)

Sequence Position	Genomic Coordinate	Motif	K-mer	Z-score	P-value
95	chr1:45976806	kkguguk	uuguggg	2.368	8.94E-03

Protein: SRSF2(Hs/Mm)

Sequence Position	Genomic Coordinate	Motif	K-mer	Z-score	P-value
90	chr1:45976811	ugcygyy	uggcguu	1.97	2.44E-02

Protein: SRSF3(Hs/Mm)

Sequence Position	Genomic Coordinate	Motif	K-mer	Z-score	P-value
16	chr1:45976885	cuckucy	gucuuac	1.98	2.39E-02
31	chr1:45976870	cuckucy	ccuuucc	1.98	2.39E-02
32	chr1:45976869	cuckucy	cuuuccu	1.98	2.39E-02
59	chr1:45976842	cuckucy	ccuuucu	2.163	1.53E-02
62	chr1:45976839	cuckucy	uucuucc	2.52	5.87E-03
145	chr1:45976756	cuckucy	aucuuuu	1.724	4.24E-02
147	chr1:45976754	cuckucy	cuuuugu	1.724	4.24E-02

Protein: SRSF5(Hs/Mm)

Sequence Position	Genomic Coordinate	Motif	K-mer	Z-score	P-value
19	chr1:45976882	yywcwsg	uuacaag	2.688	3.59E-03
37	chr1:45976864	yywcwsg	cuacagg	3.011	1.30E-03
106	chr1:45976795	yywcwsg	cuacugg	2.258	1.20E-02

Protein: TARDBP(Hs/Mm)

Sequence Position	Genomic Coordinate	Motif	K-mer	Z-score	P-value
85	chr1:45976816	ugugug	ugaguu	1.862	3.13E-02
94	chr1:45976807	ugugug	guugug	2.229	1.29E-02
96	chr1:45976805	ugugug	uguggg	2.798	2.57E-03
111	chr1:45976790	ugugug	gguuug	2.229	1.29E-02
113	chr1:45976788	ugugug	uuugua	2.229	1.29E-02
115	chr1:45976786	ugugug	uguaug	2.798	2.57E-03
122	chr1:45976779	ugugug	uguauu	1.963	2.48E-02

Protein: TRA2B(Hs/Mm)

Sequence Position	Genomic Coordinate	Motif	K-mer	Z-score	P-value
7	chr1:45976894	aaguguu	aauugug	2.234	1.27E-02
9	chr1:45976892	aaguguu	uuguggu	1.66	4.85E-02
12	chr1:45976889	aaguguu	ugguguc	1.66	4.85E-02
121	chr1:45976780	aaguguu	auguauu	1.851	3.21E-02

Protein: ZC3H14(Hs/Mm)

Sequence Position	Genomic Coordinate	Motif	K-mer	Z-score	P-value
156	chr1:45976745	uuuduuu	uuuguau	2.667	3.83E-03

Protein: ZCRB1(Hs/Mm)

Sequence Position	Genomic Coordinate	Motif	K-mer	Z-score	P-value
159	chr1:45976742	grhuuaa	guauuaa	3.325	4.42E-04
